# The transforming mutation E17K/*AKT1* is not a major event in B-cell-derived lymphoid leukaemias

**DOI:** 10.1038/sj.bjc.6604512

**Published:** 2008-07-29

**Authors:** I S Mahmoud, M A Sughayer, H A Mohammad, A A Eshtayeh, A S Awidi, M S EL-Khateeb, S I Ismail

**Affiliations:** 1 Department of Biochemistry, Faculty of Medicine, University of Jordan, Amman 11942, Jordan; 2 Department of Pathology, King Hussein Cancer Center, Amman 11941, Jordan

**Keywords:** AKT1, lymphoid leukaemia, mutation

## Abstract

Despite the major role of the AKT/PKB family of proteins in the regulation of many growth and survival mechanisms in the cell, and the increasing evidence suggesting that AKT disruption could play a key role in many human malignancies, no major mutations of *AKT* genes had been reported, until very recently when Carpten *et al* reported a novel transforming mutation (E17K) in the pleckstrin homology domain of the *AKT1* gene in solid tumours. Several laboratories are now screening for this mutation in different malignancies, and, recently, the mutation was described by Malanga *et al* in 1.9% of lung cancer patients. Considering the importance of the PI3K/AKT pathway in mediating survival and antiapoptotic signals in the B-cell types of chronic lymphocytic leukaemia (CLL) and acute lymphoblastic leukaemia (ALL), we sequenced the *AKT1* exon 3 for the above mentioned mutation in 87 specimens, representing 45 CLLs, 38 ALLs and 4 prolymphocytic leukaemia (PLL) cases, which are all of B-cell origin. Our results show that the mutation E17K/*AKT1* was not detected in the pleckstrin homology domain of AKT1 of the investigated cases. We conclude that this mutation is not a major event in B-cell-derived lymphoid leukaemias.

The v-akt murine thymoma viral oncogene homologue (AKT) is a well-established survival factor exerting variable activities, including cell survival, growth, proliferation, metabolism and glucose uptake (Nicholson and Anderson, 2002; Elstrom *et al*, 2004; Hanada *et al*, 2004; Manning and Cantley, 2007). Mounting evidence is showing the importance of AKT proteins in human malignancies (Testa and Bellacosa, 2001; Altomare and Testa, 2005; Bellacosa *et al*, 2005; Plas and Thompson, 2005). One of the first such indications was provided by the isolation of a novel transforming retrovirus ‘AKT-8’ from an AKR mouse T-cell lymphoma (Staal *et al*, 1977). This transforming retrovirus was subsequently shown to contain an intact viral oncogenic sequence called v-akt (Staal and Hartley, 1988). Since then, many studies have described perturbations of the AKT signalling pathway in multiple human cancers, which have been reviewed elsewhere (Altomare and Testa, 2005). However, mutational analysis attempted to find any genetic abnormalities of AKT kinase domains in common human malignancies revealed no major genomic alterations in the three AKT isoforms (Soung *et al*, 2006), although some reports described some minor alterations in some of the isoforms associated with specific malignancies (Robertson *et al*, 2005).

In July 2007, Carpten *et al* published a very interesting study on the *AKT1* gene in *Nature*, where they detected a novel transforming mutation involving a glutamic acid (E) to lysine (K) substitution at amino acid 17 (E17K) in the pleckstrin homology domain of the *AKT1* gene. This E17K point mutation was identified in 8% of breast cancers, 6% of colorectal cancers and 2% of ovarian cancers (Carpten *et al*, 2007). Screening studies to find this novel mutation in other cancers were carried out by different investigators in the past few months. Although Tibes *et al* (2008) and Schüller *et al* (2008) did not find the mutation in acute myeloid leukaemia, and glioblastomas and medulloblastomas, respectively, the mutation was detected in 1.9% of lung cancers; where it represented 5.5% of the squamous cell carcinoma histotype (Malanga *et al*, 2008).

Chronic lymphocytic leukaemia (CLL) is the most common type of leukaemia in older adults in the West (Inamdar and Bueso-Ramos, 2007). Chromosome defects are the most common genetic abnormalities seen in CLL, and the last decades have seen much progress in delineating the role of these genetic aberrations in the pathogenesis of CLL (Cotter and Auer, 2007). However, the genetics of CLL is still not fully understood. The PI3K/AKT pathway has been shown to play a central role in promoting cell survival and growth of B-cell CLL (Barragán *et al*, 2002, 2006; Cuní *et al*, 2004; Petlickovski *et al*, 2005; Longo *et al*, 2007). Also, in a recent study, Longo *et al* (2008) described the critical role of AKT in mediating the antiapoptotic signals along with Mcl-1 downstream of the B-cell receptor in CLL. Similarly, AKT has been described to mediate survival of precursor B-acute lymphoblastic leukaemia (pre-B-ALL) cells through activation of RAFTK, which plays an antiapoptotic role (Sarkar *et al*, 2002). Also, Wang *et al* (2004) have showed the pivotal role of Akt in mediating stromal cell regulation of ALL cell apoptosis.

Considering the central role of AKT in CLL and ALL, we performed a screening study in an attempt to detect the mutation E17K/*AKT1* in these types of haematological malignancies.

## Patients, materials and methods

### Patient samples

A total of 87 EDTA–blood samples (45 CLLs, 38 ALLs and 4 prolymphocytic leukaemias (PLLs)) were collected from Jordanian patients attending King Hussein Cancer Center and Jordan University hospital, according to protocols approved by the Institutional Review Board. Informed consent was obtained from each patient.

The CLL specimens represented all stages of the Rai staging system (Rai *et al*, 1975), which was available for 41 out of 45 patients, whereas all ALL and PLL specimens were of the B-cell type (Table 1).

### Materials and methods

DNA was extracted from whole-blood samples using the Wizard genomic DNA purification kit (Promega, Madison, WI, USA). Polymerase chain reaction (PCR) was used to amplify genomic DNA encompassing the coding sequences and intronic borders of exon 3 of the *AKT1* gene in patients' DNA. The primers used for PCR were the same as those used by Carpten *et al* (2007) (F: 5′-ACATCTGTCCTGGCACAC-3′, R: 5′-GCCAGTGCTTGTTGCTTG-3′).

The PCR was performed in 50 μl reaction containing 200 ng of genomic DNA, 200 μM dNTPs, 1.5 mM MgCl_2_, 1 U Go *Taq* polymerase (Promega) and 10 pmol of each primer (Invitrogen, Carlsbad, CA, USA). The reaction conditions were as follows: initial denaturation of 3 min at 95°C, followed by 34 cycles of 30 s at 95°C, 30 s at 52°C, 1 min at 72°C and a final extension of 6 min at 72°C. The PCR products were purified using the Wizard SV gel and PCR cleanup system (Promega) and then sequenced using the same PCR primers. The sequencing reactions were performed at Macrogen Inc., (Seoul, South Korea) using the Big Dye™ terminator cycle sequencing kit (Applied Biosystems, Foster Ctiy, CA, USA) and the ABI 3730 XL analyzer (Applied Biosystem).

## Results and discussion

The sequencing analysis for exon 3 of the human *AKT1* gene in 87 samples (45 CLLs, 38 pre-B-cell ALLs and 4 B-cell PLLs) revealed the absence of the point mutation G>A at nucleotide 49 (E17K), which was first identified by Carpten *et al* (2007).

The genetic abnormalities seen in CLL differ from most other forms of haematological malignancies, including ALL. Although CLL is usually characterised by chromosomal deletions (Cotter and Auer, 2007), ALL and many other forms of leukaemia usually harbour chromosomal translocations in a significant number of transformed cells. Despite the extensive sequencing analysis over the last decade, no major point mutations have been identified in CLL or ALL.

Because of the oncogenic potential of the mutation E17K, and its ability to specifically induce B-cell leukaemia in mice (Carpten *et al*, 2007), in addition to the importance of AKT proteins in mediating survival and antiapoptotic signals in B-cell lineages of CLL and ALL, we sought to look for this mutation in B-cell lymphoid leukaemia patients. The CLL patients who were included in this study represented the stable or advanced stages of the disease; all ALL patients were of the pre-B-cell type and four patients with PLL were also included.

From our findings we conclude that the mutation E17K/*AKT1* is unlikely to cause major transforming activity in B-cell-origin CLL, ALL and PLL. However, given the small size of the sample analysed, another large-scale study could increase the statistical power of our results. Also, we did not include the T-lineage cells of ALL in this study, which is something we will be working on next, as it is worthwhile to screen for the mutation in this subclass of acute leukaemia.

This report assesses the presence of E17K/*AKT1* in B-cell lymphoid leukaemias, including the chronic and acute phases. Our results are supported by two recent reports, which were published while preparing this paper, by Kim *et al* (2008) and Zenz *et al* (2008), who also came to a similar conclusion regarding the significance of E17K/*AKT1* in B-cell ALL and CLL, respectively. Further work is needed to investigate the possibility of analogous mutations in other AKT isoforms as well as to detect the status of this mutation in other related haematological malignancies.

## Figures and Tables

**Table 1:** Characteristics of patient samples

	**Patients**		
		**Sex**	
	***n*** a	**M**	**F**
*CLL (Rai stage)*			
0	8	5	3
I	8	8	—
II	8	6	2
III	9	8	1
IV	8	3	5
*ALL*			
*Pre*-B	38	21	17
*B-PLL*	4	3	1

ALL=acute lymphoblastic leukaemia; B-PLL=B-cell prolymphocytic leukaemia; CLL=chronic lymphocytic leukaemia; pre-B=precursor B-cell.

Rai stages were available for 41 out of 45 CLL patients.

a Number of samples.
